# Comparing the Differential Diagnostic Values of ^18^F-Alfatide II PET/CT between Tuberculosis and Lung Cancer Patients

**DOI:** 10.1155/2018/8194678

**Published:** 2018-02-19

**Authors:** Xiaoqing Du, Yu Zhang, Liping Chen, Baoming Mi, Qingjun You, Yuping Xu, Donghui Pan, Weixing Wan, Min Yang, Chunjing Yu

**Affiliations:** ^1^Department of Nuclear Medicine, Affiliated Hospital of Jiangnan University (Wuxi No. 4 People's Hospital), Wuxi, China; ^2^Key Laboratory of Nuclear Medicine, Ministry of Health, Jiangsu Institute of Nuclear Medicine, Wuxi, China

## Abstract

**Purpose:**

To compare the differential diagnostic values of ^18^F-Alfatide II PET/CT between tuberculosis and lung cancer patients and in patients with sarcoidosis and common inflammation.

**Methods:**

Nine inflammation patients (4 tuberculosis, 3 sarcoidosis, and 2 common inflammation) and 11 lung cancer patients were included in this study. All patients underwent ^18^F-FDG and ^18^F-Alfatide II PET/CT within 2 weeks, followed by biopsy and surgery. The maximized standard uptake value (SUVmax) and the mean standard uptake value (SUVmean) were evaluated.

**Results:**

The active tuberculosis lesions showed a high accumulation of ^18^F-FDG, but varying degrees of accumulation of ^18^F-Alfatide II, including negative results. The SUVmax of ^18^F-Alfatide II in malignant lesions was significantly higher than that in tuberculosis (4.08 ± 1.51 versus 2.63 ± 1.34, *P* = 0.0078). Three patients with sarcoidosis showed negative results in ^18^F-Alfatide II PET/CT.

**Conclusions:**

The expression of *α*_V_*β*_3_ is much lower in tuberculosis as compared to that in lung cancer, and accumulation of ^18^F-Alfatide II varied even in lesions of the same patient. The negative results of sarcoidosis patients led to the speculation that *α*_V_*β*_3_ was not expressed in those lesions.

## 1. Introduction

Lung cancer is one of the largest malignant tumors with fast-growing morbidity and mortality. ^18^F-FDG PET/CT has been verified as a crucial tool for detecting, identifying, and staging lung cancer. However, the specificity of ^18^F-FDG PET/CT in lung cancer is controversial as some benign lesions such as tuberculosis and sarcoidosis also show a high accumulation of ^18^F-FDG. Thus, a new tracer with higher specificity in differentiating lung cancer and inflammation is essential.

The expression of integrin *α*_V_*β*3 on the surface of cancer cells and neovascularization endothelial cells is upregulated in cancer, inflammation, and wound [1]. ^18^F-Alfatide II is an annular dimer RGD (Arg-Gly-Asp) tracer targeting integrin *α*_V_*β*3. Previous studies reported that the uptake of Alfatide in lung cancer and tuberculosis lesions is markedly different [2, 3]. Thus, additional clinical data is needed to illustrate whether this new tracer is beneficial for the differentiation of tuberculosis from lung cancer.

The present study investigated the differential diagnostic value of ^18^F-Alfatide II PET/CT between tuberculosis and lung cancer patients. Also, the angiogenesis in sarcoidosis and chronic inflammations was investigated.

## 2. Materials and Methods

### 2.1. Radiopharmaceutical Preparation

The kit was provided by Jiangsu Institute of Nuclear Medicine. Synthesis of ^18^F-Alfatide II has been described previously [4].

### 2.2. Patients

The local institutional review board approved the ^18^F-Alfatide II PET/CT compliment protocol. Written informed consent was obtained from each patient. The cohort consisted of 9 patients [5 men and 4 women; aged 25–71 (55 ± 16) years] with suspected inflammations and 11 patients [10 men, 1 women; aged 48–78 (66 ± 9) years] with suspected lung cancer.

### 2.3. PET/CT Acquisition and Image Analysis

^18^F-FDG and ^18^F-Alfatide II PET/CT were performed at an interval of 2 weeks. Patients were required to fast at least 6 h before ^18^F-FDG (5.55 MBq/kg) intravenous injection. The acquisitions were conducted at 60 min after the injection. The patients were placed in a supine position on the scanner bed. Imaging data were acquired from the skull to the thigh, using PET/CT scanner, at 1.5 min/bed position. Low-dose CT was performed for attenuation correction and lesion localization. ^18^F-Alfatide II PET/CT was performed on the next day without any specific preparation before the examination. ^18^F-Alfatide II (248.27 ± 45.14 MBq) was injected intravenously in all patients. The acquisition procedure and parameters were identical to that as ^18^F-FDG PET/CT. Regions of interest (ROIs) were drawn manually on the site of lesions based on the corresponding CT images.

PET/CT Scanner was from Siemens (Biograph True Point PET/CT).

Visual analysis was used to evaluate the preliminary accumulation of ^18^F-Alfatide II and ^18^F-FDG in tuberculosis and lung cancer. Maximum standard uptake value (SUVmax), mean standard uptake value (SUVmean), and lesion/muscle (L/M) ratio recorded the uptake of the lesions in this study. The uptake of the right hip muscle was selected as a reference for lesions. All lesions were divided into different regions of head-neck, chest, abdomen, and pelvis. The largest lesion of each region was chosen to measure the uncountable lesions.

Two physicians evaluated the images independently, and the discrepancies were resolved by consultation.

### 2.4. Pathological Analysis and Follow-Up

*Inflammation Group*. Number 1 patient was confirmed as thoracic tuberculosis by biopsy. Number 2–4 patients, receiving PPD test, T-SPOT test, and antituberculosis treatment, were followed up for 16, 17, and 33 months, respectively; they were confirmed as lung tuberculosis, lymph node tuberculosis, and lung tuberculosis mixed with tuberculous pleurisy. Number 5 patient was confirmed as sarcoidosis by bronchoscope puncture biopsy. Number 6-7 patients, receiving no treatment and followed up for 17 and 28 months, respectively, were diagnosed as sarcoidosis. Number 8 patient was shown to have chronic inflammation accompanied by fibrosis as assessed by percutaneous lung biopsy. Number 9 patient was diagnosed with inflammation caused by common infection after 20 months' follow-up based on CT (Table 1).

*Malignancy Group*. Eight patients received surgery. One patient did not undergo surgery since the pulmonary trunk was invaded by cancer, and two patients were not recommended surgery as distant metastasis detected by PET/CT and MRI. All patients were confirmed by pathology; one distant metastasis patient received bronchoscopy biopsy, while the other underwent clavicle lymph node excision (Table 2).

### 2.5. Statistical Analysis

Mean ± standard deviation (m ± SD) was used to express all quantitative data. Differences in SUVmax/SUVmean between patients with different diseases were compared and assessed by *t*-test or Mann–Whitney *U* test. All statistical analyses were carried out using SAS 9.2. *P* < 0.05 indicated statistical significance.

## 3. Results

### 3.1. Safety

Patients did not report any subjective effects following the injected dose of ^18^F-Alfatide II. No adverse events were noted during the examination of ^18^F-Alfatide II PET/CT or follow-up (at least 6 months).

### 3.2. Visual Analysis Results

**Figure 1:**
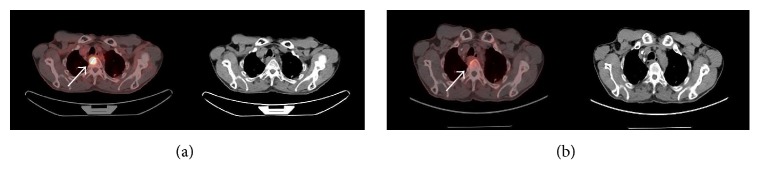
^18^F-FDG (a) and ^18^F-Alfatide II (b) PET/CT images of a thoracic tuberculosis patient. T2, T3, and T4 showed intense ^18^F-FDG uptake and mild ^18^F-Alfatide II uptake. The white arrows indicate tuberculosis lesions in T4.

**Figure 2:**
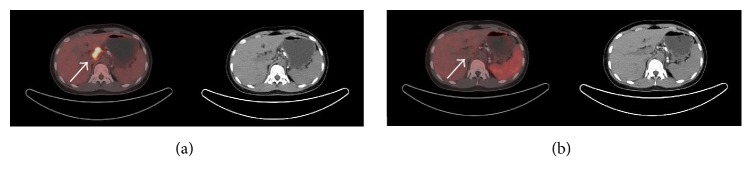
^18^F-FDG (a) and ^18^F-Alfatide II (b) PET/CT images of a lymph node tuberculosis patient. Lymph nodes tuberculosis lesions showed intense ^18^F-FDG uptake and no ^18^F-Alfatide II uptake. The white arrows indicate tuberculosis lesions in porta hepatis lymph nodes.

**Figure 3:**
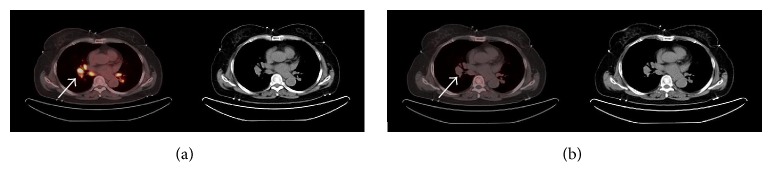
^18^F-FDG (a) and ^18^F-Alfatide II (b) PET/CT images of a sarcoidosis patient. All lesions showed intense ^18^F-FDG uptake and no ^18^F-Alfatide II uptake. The white arrows indicate sarcoidosis lesions in mediastinum and hilus pulmonis.

The accumulation of ^18^F-Alfatide II in tuberculosis was much lower than that of ^18^F-FDG (Figures 1 and 2), while no accumulation was observed in sarcoidosis lesions (Figure 3). Additionally, 2 chronic inflammations showed a high accumulation of ^18^F-Alfatide II.

All lung cancer patients in this study showed a high accumulation of ^18^F-Alfatide II, including brain and bone metastasis. The results were similar to that in the previous reports [5].

### 3.3. Preliminary Diagnostic Value of ^18^F-FDG PET/CT and ^18^F-Alfatide II PET/CT in Tuberculosis

The SUVmax of tuberculosis was calculated as 7.53 ± 2.88 and 2.63 ± 1.34 for ^18^F-FDG and ^18^F-Alfatide II, respectively. The SUVmean of tuberculosis was 4.58 ± 1.73 and 1.86 ± 1.0, respectively.

### 3.4. Preliminary Diagnostic Value of ^18^F-FDG PET/CT and ^18^F-Alfatide II PET/CT in Chronic Inflammation

The SUVmax of two patients with chronic inflammation was 10.80 and 1.62 for ^18^F-FDG and 9.13 and 5.75 for ^18^F-Alfatide II, respectively. The SUVmean was 5.33, 0.99 and 5.12, 2.65, respectively.

### 3.5. Preliminary Diagnostic Value of ^18^F-FDG PET/CT and ^18^F-Alfatide II PET/CT in Sarcoidosis

The SUVmax of ^18^F-FDG and ^18^F-Alfatide II in sarcoidosis was calculated as 8.82 ± 5.17 and 1.77 ± 0.69, respectively, while SUVmean was 5.34 ± 3.08 and 1.28 ± 0.63, respectively.

### 3.6. Preliminary Diagnostic Value of ^18^F-FDG PET/CT and ^18^F-Alfatide II PET/CT in Lung Cancer

The SUVmax of ^18^F-FDG and ^18^F-Alfatide II in lung cancer was 12.04 ± 4.67 and 4.08 ± 1.51, respectively, while SUVmean was 4.55 ± 1.98 and 1.99 ± 0.81, respectively.

### 3.7. Difference between the SUVmax of Malignant Lesions and Tuberculosis Lesions

The lesion-to-lesion analysis showed that the SUVmax of ^18^F-Alfatide II in malignant lesions was 4.08 ± 1.51, which was significantly higher than that in tuberculosis (2.63 ± 1.34, *P* = 0.0078).

### 3.8. Difference between the SUVmean of Malignant Lesions and Tuberculosis Lesions

The SUVmean of ^18^F-Alfatide II in malignant lesions was 1.99 ± 0.81 without any statistically significant difference from that of tuberculosis (1.86 ± 1.0, *P* = 0.3820).

### 3.9. Preliminary Diagnostic Value of ^18^F-FDG and ^18^F-Alfatide II PET/CT in Inflammations (Active TB, Chronic Inflammation, and Sarcoidosis) and Lung Cancer

See Table 3.

## 4. Discussion

Integrin *α*_V_*β*_3_ is overexpressed not only in various tumor cells and tumor neovasculature [6] but also in chronic inflammatory diseases, such as inflammatory bowel disease (IBD) and rheumatoid arthritis (RA) [7, 8]. Previous studies reported that angiogenesis and chronic inflammation are interrelated [7, 9]. Jackson et al. [10] suggested that several resident cells (fibroblasts, monocytes-macrophages, neutrophils, and lymphocytes) can promote angiogenesis when the microenvironment becomes hypoxic or inflammatory, thereby facilitating the migration of inflammatory cells to inflammatory sites and the supply of nutrients and oxygen to the proliferating tissue. The frequent dual functionality of angiogenic factors such as *α*_V_*β*_3_ and VEGF reflects the close relationship between angiogenesis and inflammation [11]. Cao et al. [12] demonstrated specific uptake of *α*_V_*β*_3_ in the chronic inflammation of mouse ear using ^64^Cu-DOTA-RGD tetramer PET imaging.

Chin et al. [13] carried out a PET imaging study with ^18^F-FPP(RGD)_2_ in a healthy woman volunteer, and no unusual or adverse patient symptoms were found on the day of imaging as well as during follow-up. Wan et al. [2] did not record any adverse events associated with ^18^F-Alfatide in the first subject during the study, in which nine lung cancer patients were investigated. No adverse events occurred in all patients during or after the ^18^F-Alfatide II PET/CT imaging in the current study. All these investigations revealed that using RGD tracers labeled by ^18^F is safe for patients.

Tuberculosis is a major global health problem with an estimated 8.6 million new cases worldwide in 2012 [14]. The tuberculous granuloma is an organized collection of differentiated macrophages surrounded by T-lymphocytes, B-lymphocytes, dendritic cells, fibroblasts, and extracellular matrix components [15]. Some studies demonstrated *α*_V_*β*_1_ expression in lung granulomas and lymph nodes of sarcoid patients [16]. Rojas et al. [17] found that the mycobacterial glycolipid phosphatidylinositol mannoside interacts directly with *α*_V_*β*_1_ integrin VLA-5 on CD4^+^ T-lymphocytes, resulting in fibronectin binding and T-cell migration. Several studies have reported increased levels of VEGF in granulomatous disease, such as pulmonary tuberculosis [18–21] and Crohn's disease [22]. Hur et al. [23] reported that median concentration of serum VEGF-A was significantly higher in tuberculosis patients than that in the latent tuberculosis infection and control groups. Four patients with active tuberculosis showed varying degree of accumulation of ^18^F-Alfatide II in this study, including negative results, and even the positive lesions showed a low accumulation of ^18^F-Alfatide II than that of ^18^F-FDG. Kang et al. [3] reported that tuberculosis granuloma and the surrounding vasculature epithelium showed baseline *α*_V_*β*_3_ expression as assessed by immunohistochemistry. The diversity in the current study revealed different expression of *α*_V_*β*_3_ in all tuberculosis lesions.

All lung cancer lesions and the metastases in the brain and bone showed an increased RGD uptake. A significant difference was noted in SUVmax between the lung cancer and tuberculosis groups, which indicated that RGD PET/CT might differentiate lung cancer from tuberculosis.

Sarcoidosis is an immunological, granulomatous disorder affecting multiple systems. The presence of noncaseating granulomas in involved organs is a pathological feature [24]. The common sites of the disease are lung, mediastinum, and hilus pulmonis lymph node [25, 26]. The precise pathogenesis is yet unknown, which might include various factors: environmental, occupational exposure, the presence of infectious agents, and genetic susceptibility [27–29]. Various studies suggested that angiogenic factors contribute to the pathogenesis of sarcoidosis [30, 31]. Agostini et al. [32] and Antoniou et al. [31] indicated the presence of angiogenesis in the pathogenesis of granulomatous and pulmonary fibrosis. Tzouvelekis et al. [33] revealed an abundant expression of VEGF and ING4 within the granulomatous tissue, localized in the epithelioid and giant cells. Three sarcoidosis patients, in this study, showed negative results in ^18^F-Alfatide II PET/CT, thereby indicating the lack of *α*_V_*β*_3_ expression in sarcoidosis. Kambouchner et al. [34] proposed the presence of an avascular microenvironment within sarcoid lesions. Tzouvelekis et al. [33] speculated that abundant expression of VEGF might be implicated in the inflammatory than the angiogenic cascade of sarcoidosis. Murdoch et al. supported this speculation with respect to the pleiotropic properties of VEGF in promoting the Th1-dependent immunity via facilitation of monocyte recruitment and T-cell migration to sites of ongoing inflammation [35]. The results from the current study were in agreement with the theory by Kambouchner et al. and Tzouvelekis et al.

Furthermore, the present study comprised 2 common infection patients: one patient showed high accumulation of ^18^F-Alfatide II as well as ^18^F-FDG, while the other showed a high accumulation of ^18^F-Alfatide II compared to ^18^F-FDG. Winter et al. [36] speculated that integrin *α*_V_*β*_3_ was a potential marker of inflammation and angiogenesis in atherosclerotic lesions. Srivatsa et al. and Hansson both observed persistently high levels of *α*_V_*β*_3_ expression between 7 and 21 days following injury in the neointima, media, and adventitia [37, 38]. Other studies demonstrated the expression of integrin *α*_V_*β*_3_ on activated macrophages by different methods [39–41]. When acute inflammation transforms into subacute and chronic inflammation, macrophages are gradually increased in number in lesions than the neutrophils. Thus, the high accumulation of ^18^F-Alfatide II in the 2 patients in this study indicated the chronic inflammatory stage, which was confirmed by fibrosis tested by percutaneous lung biopsy in one lesion. Storgard et al. [42] reported that the treatment with cyclic RGD peptide c(RGDfV), an integrin *α*_V_*β*_3_ antagonist, significantly inhibited the disease progression in an experimental RA model. Taken together, ^18^F-Alfatide II PET/CT may not only detect chronic inflammation but also allow the evaluation of angiogenesis and neovascularization during chronic inflammation and guide the selection of patients for antiangiogenesis therapy.

Nevertheless, the present study has some deficiencies. (1) Early experimental design was not perfect; for example, we did not recruit lymphoma patients to compare with sarcoidosis in ^18^F-Alfatide II PET/CT. (2) The number of patients was small. (3) Further investigations are essential.

## 5. Conclusion

The accumulation of lung cancer and tuberculosis exhibits distinct difference, which might be valuable in differentiating the two diseases. Three sarcoidoses showed negative results, and thus, we speculated the lack of *α*_V_*β*_3_ expression within sarcoidosis. ^18^F-Alfatide II might be valuable in the evaluation of angiogenesis and neovascularization during chronic inflammation, which could guide the selection of patients for antiangiogenesis therapy and evaluate the clinical effect of the treatment.

## Figures and Tables

**Table 1:** Patient demographics (inflammation group).

Patient number	Age (years)	Sex	Histology
1	69	M	Thoracic tuberculosis
2	71	M	Lung tuberculosis
3	25	F	Lymph node tuberculosis
4	63	M	Lung tuberculosis + tuberculous pleurisy
5	66	F	Sarcoidosis
6	56	F	Sarcoidosis
7	32	F	Sarcoidosis
8	67	M	Chronic-inflammation with fibrosis
9	66	M	Common inflammation

**Table 2:** Patient demographics (malignancy group).

Patient number	Age (years)	Sex	Histology
1	65	M	Squamous cell carcinoma (SCC)
2	75	M	Adenocarcinoma
3	78	M	Squamous cell carcinoma (SCC)
4	72	M	Squamous cell carcinoma (SCC)
5	58	M	Squamous cell carcinoma (SCC)
6	57	M	Squamous cell carcinoma (SCC)
7	67	M	Squamous cell carcinoma (SCC)
8	48	M	Squamous cell carcinoma (SCC)
9	57	F	Adenocarcinoma
10	75	M	Adenocarcinoma
11	74	M	Adenocarcinoma

**Table 3:** SUVmax and SUVmean of lesions.

	^18^F-FDG	^18^F-Alfatide II	^18^F-FDG	^18^F-Alfatide II
	SUVmax	SUVmax	SUVmean	SUVmean
Tuberculosis	7.53 ± 2.88	2.63 ± 1.34	4.58 ± 1.73	1.86 ± 1.0
Lung cancer	12.04 ± 4.67	4.08 ± 1.51	4.55 ± 1.98	1.99 ± 0.81
Sarcoidosis	8.82 ± 5.17	1.77 ± 0.69	5.34 ± 3.08	1.28 ± 0.63
Chronic inflammation 1	10.80	9.13	5.33	5.12
Chronic inflammation 2	1.62	5.75	0.99	2.65
